# The Mutational Robustness of Influenza A Virus

**DOI:** 10.1371/journal.ppat.1005856

**Published:** 2016-08-29

**Authors:** Elisa Visher, Shawn E. Whitefield, John T. McCrone, William Fitzsimmons, Adam S. Lauring

**Affiliations:** 1 Division of Infectious Diseases, Department of Internal Medicine, University of Michigan, Ann Arbor, Michigan, United States of America; 2 Department of Microbiology and Immunology, University of Michigan, Ann Arbor, Michigan, United States of America; Imperial College London, UNITED KINGDOM

## Abstract

A virus’ mutational robustness is described in terms of the strength and distribution of the mutational fitness effects, or MFE. The distribution of MFE is central to many questions in evolutionary theory and is a key parameter in models of molecular evolution. Here we define the mutational fitness effects in influenza A virus by generating 128 viruses, each with a single nucleotide mutation. In contrast to mutational scanning approaches, this strategy allowed us to unambiguously assign fitness values to individual mutations. The presence of each desired mutation and the absence of additional mutations were verified by next generation sequencing of each stock. A mutation was considered lethal only after we failed to rescue virus in three independent transfections. We measured the fitness of each viable mutant relative to the wild type by quantitative RT-PCR following direct competition on A549 cells. We found that 31.6% of the mutations in the genome-wide dataset were lethal and that the lethal fraction did not differ appreciably between the HA- and NA-encoding segments and the rest of the genome. Of the viable mutants, the fitness mean and standard deviation were 0.80 and 0.22 in the genome-wide dataset and best modeled as a beta distribution. The fitness impact of mutation was marginally lower in the segments coding for HA and NA (0.88 ± 0.16) than in the other 6 segments (0.78 ± 0.24), and their respective beta distributions had slightly different shape parameters. The results for influenza A virus are remarkably similar to our own analysis of CirSeq-derived fitness values from poliovirus and previously published data from other small, single stranded DNA and RNA viruses. These data suggest that genome size, and not nucleic acid type or mode of replication, is the main determinant of viral mutational fitness effects.

## Author Summary

Like other RNA viruses, influenza virus has a very high mutation rate. While high mutation rates may increase the rate at which influenza virus will adapt to a new host, acquire a new route of transmission, or escape from host immune surveillance, data from model systems suggest that most new viral mutations are either lethal or highly detrimental. Mutational robustness refers to the ability of a virus to tolerate, or buffer, these mutations. The mutational robustness of a virus will determine which mutations are maintained in a population and may have a greater impact on viral evolution than mutation rate. We defined the mutational robustness of influenza A virus by measuring the fitness of a large number of viruses, each with a single point mutation. We found that the overall robustness of influenza was similar to that of poliovirus and other viruses of similar size. Interestingly, mutations appeared to be more easily accommodated in hemagglutinin and neuraminidase than elsewhere in the genome. This work will inform models of influenza evolution at the global and molecular scale.

## Introduction

The predictable burden of seasonal influenza and the unpredictability of the next pandemic are attributable in large part to the rapid evolution of influenza virus [1–4]. Like other RNA viruses, influenza viruses replicate with extremely low fidelity, with a mutation rate of roughly 2 x 10^−5^ substitutions per nucleotide copied per cellular infection [5–7]. Influenza viruses also undergo reassortment of their genomic segments, a combinatorial exchange of genetic material analogous to recombination in other RNA viruses [8,9]. Together, low replicative fidelity and frequent reassortment allow influenza virus populations to generate significant diversity. This capacity may allow influenza viruses to maintain, or to quickly generate, the requisite mutations that mediate cross species transmission, escape from neutralizing antibody, or drug resistance [10].

The focus on mutation as a driving force in viral evolution has tended to downplay the tremendous fitness cost of mutation [11,12]. Here, we define viral fitness as the capacity of an individual, or population, to generate infectious progeny. Most mutations have deleterious effects on fitness, which suggests that mutational tolerance may play a significant role in determining the genetic diversity that can be maintained within a population [13]. Mutational robustness refers to phenotypic stability in the face of mutation [14–16]. High mutation rates select for increased mutational robustness [17], and a more robust population can increase its genetic diversity without a dramatic alteration in mean fitness. A virus’ intrinsic robustness may influence its fitness *in vitro* and virulence *in vivo* [18].

The impact of individual mutations on viral fitness is the mutational fitness effect (MFE). A virus’ mutational robustness is described in terms of the strength and distribution of the MFE [19]. Together with mutation rate, the MFE governs many aspects of evolution including: the relative importance of selection vs. genetic drift, the efficiency of selection and adaptation, the impact of recombination (or reassortment), and the role of epistasis in fixing new and beneficial mutations. It is therefore essential for accurate models of molecular evolution [20,21]. A virus’ sensitivity to mutation may also determine the effectiveness of lethal mutagenesis [22–24].

Early studies of mutational fitness effects relied on mutation accumulation (MA) experiments, where the imposition of extreme bottlenecks propagates and fixes newly generated mutations by drift as opposed to selection [25–29]. An alternative approach commonly used in RNA viruses is to accelerate mutation accumulation by passaging virus in the presence of mutagenic drugs [30–32]. While both methods provide valuable information about the average fitness impact of random mutation, uncertainty about the number of mutations per clone and the fitness effect of each individual mutation makes it difficult to accurately model a distribution. These issues also complicate more recent, high throughput methods based on next generation sequencing [33–35]. Furthermore, none of these approaches provide accurate estimates of the fraction of mutations that are lethal to the virus.

A less exhaustive, but more controlled assay for MFE is to measure the fitness of a set of viral clones, each with a randomly selected single nucleotide substitution. This approach unambiguously assigns fitness values, or selection coefficients, to individual mutations. It is also highly quantitative and allows for estimation of the lethal fraction. The first such study, of vesicular stomatitis virus (VSV), found that over 90% of random single nucleotide mutations reduce replicative fitness and 40% are lethal in this negative sense RNA virus [13]. Subsequent work by Sanjuan, Elena, and colleagues found somewhat similar distributions of MFE in f1 (ssDNA phage), phiX 174 (ssDNA phage), QB (+ssRNA phage), and tobacco etch virus (+ssRNA virus) [19,36–40]. Together these data suggest that despite their differences in genome organization and replication strategy, ssRNA and ssDNA viruses are equally sensitive to mutation.

Despite their importance to pathogen evolution, all available genome-wide studies of viral MFE have been performed in evolutionary model systems. Here we characterize the distribution of MFE in influenza A virus, a segmented negative sense RNA virus whose evolutionary dynamics are important to global health. We used site directed mutagenesis to generate a library of plasmids encoding 128 influenza A viruses, each with a single point mutation in the A/WSN33/H1N1 genetic background. The large number of mutants allowed us to define the MFE across the genome and to compare the lethal fraction and MFE between segments coding for the surface proteins to those coding for the internal proteins. We find that the MFE of influenza A are remarkably similar to those of other viruses with varying genome structure. While similar proportions of mutations in the surface proteins and internal proteins were lethal, the average impact of mutation appeared to be less deleterious in HA and NA. Our results suggest that the size and compactness of a virus’ genome, and not necessarily the genomic nucleic acid or mode of replication, are major determinants of its intrinsic mutational robustness.

## Results

Our primary goal was to determine the distribution of mutational fitness effects across the influenza A genome. We generated all single nucleotide mutations in the commonly used laboratory strain, A/WSN/33/H1N1, hereafter referred to as WSN33 or wild type (WT) [41]. In WSN33, the 8 genomic segments range in size from 0.9 to 2.3 kb. We planned to make 149 mutants and grouped them into two libraries–“genome-wide” and “comparison”–of pre-specified size and composition. Of the 149 total mutations we attempted (S1 Table), we successfully generated 128 (86%). For our “genome-wide” library, we reasoned that in order to achieve an unbiased distribution of mutations throughout the genome, our library should contain a number of mutations on each segment that is proportional to the size of each segment—PB2 17.2%, PB1 17.2%, PA 16.4%, HA 13.1%, NP 11.5%, NA 10.4%, M 7.5%, NS 6.5%, of the total genome respectively. We used a custom R script to choose randomly the nucleotide position and substitution type in accordance with this distribution. For our unbiased genome-wide analysis (n = 95), we generated 14 (14.7%) PB2, 16 (16.8%) PB1, 16 (16.8%) PA, 14 (14.7%) HA, 12 (12.6%) NP, 10 (10.5%) NA, 8 (8.4%) M and 5 (5.3%) NS mutations. Though we failed to generate 14% of attempted mutations, this did not significantly alter the distribution of the mutations across the eight segments (S1A Fig).

Viral surface proteins that are targeted by the immune system often exhibit greater sequence diversity than internal structural and enzymatic proteins. In many cases, the relationship of this diversity to the intrinsic mutational robustness of the genes encoding these surface proteins is unknown. Therefore, a secondary goal of our study was to compare the distribution of mutational fitness effects for the HA- and NA-encoding segments to the other 6 segments, which code for internal proteins. We improved our power to detect a difference by generating an additional 18 HA and 15 NA mutations. In this aggregate “comparison” library (n = 128), 45% (n = 57) of our library is contained on segments coding for HA and NA and 55% (n = 71) on the other 6 segments. (S1B Fig). The entire data set includes 38 transition mutations and 90 transversion mutations, which is in the range of what one would expect by chance (Fisher test, p = 0.75).

### Identification of lethal mutations

Previous work indicates that a substantial, but varying proportion, of mutations in RNA viruses are lethal. Accurate assessment of this lethal fraction is an essential, and non-trivial, task. In a reverse genetic approach, the efficiency of transfection and viral recovery are key parameters, since a failed transfection and a lethal mutation will both give supernatants with undetectable titers. We used two approaches to address this problem. In the first, we estimated the transfection failure rate and calculated the probability of miscalling a viable mutant as lethal. Over the course of this study, we transfected the WT virus 19 individual times, and never failed to recover virus in our P0 supernatants. As in [13], we quantified the expected probability of a transfection failure as if we had observed a single WT transfection failure. This conservative approach gave a per transfection failure rate of < 5.26%. Because we attempted three independent transfections for each candidate lethal, the likelihood of repeated transfection failure is 0.0526^3^, or 1.46 x10^-4^. In a set of 128 viruses, we would expect to falsely identify fewer than one (128 * 1.46 x 10^−4^ = 0.019) virus as lethal. This probabilistic model assumes equal transfection efficiency for WT and mutant viruses. We also tested one of our mutants, PB2_14 (PB2 C532A), which had moderately reduced fitness (0.81, see below). Here too, we successfully recovered virus in 19 individual transfections.

We also observed several cases of mutants with undetectable titers at P0 and a moderate P1 titer after blind passage of the supernatant from transfected cells. We characterized these P1 stocks by next generation sequencing, and in all cases, found either reversion of the introduced mutation or contamination of the culture by other viruses that were transfected or passaged on the same day. We therefore considered a mutation to be lethal if we had undetectable virus in three independent transfections or if we were unable to recover the mutation in a P1 stock after blind passage of the P0 stock. Using these criteria, there were 30 lethal mutations (31.6%) in the genome-wide dataset and 38 lethal mutations in the combined dataset (29.7%, Table 1). The lethal fraction did not differ appreciably between either the HA/NA encoding segments and the rest of the genome (16/57 vs. 22/71; Fisher exact test, p = 0.85) or the HA encoding segment and the rest of the genome (7/32 vs. 31/96; Fisher exact test, p = 0.37). We had 62% to detect a two fold difference in the lethal fraction for HA/NA and 52% power to detect a two fold difference in the lethal fraction for HA. No synonymous mutations were lethal, but 2 non-coding mutations and a stop codon loss were lethal.

**Table 1:** Lethal mutations.

Segment	Mutation	Amino Acid	Dataset	Clone ID
1 (PB2)	C839A	T271K	Genomewide	PB2-10
	U1559G	V511G	Genomewide	PB2-2
	U2305C	Stop reversion	Genomewide	PB2-1
2 (PB1)	U675A	Y217stop	Genomewide	PB1-3
	A728U	K235M	Genomewide	PB1-15
	C1123U	Q367stop	Genomewide	PB1-14
	U1232G	L403W	Genomewide	PB1-9
	U1268G	L415stop	Genomewide	PB1-16
	G1581U	E519D	Genomewide	PB1-7
3 (PA)	A263U	E80V	Genomewide	PA-12
	G529C	A169P	Genomewide	PA-11
	C1324A	P434T	Genomewide	PA-14
	G1634C	W537S	Genomewide	PA-5
	C1682A	A553D	Genomewide	PA-4
4 (HA)	U55G	L8R	Genomewide	HA-11
	U799G	I256R	Genomewide	HA-12
	U915C	C295R	HA/NA	HA-25
	C1137A	H369N	HA/NA	HA-32
	A1143U	N371Y	Genomewide	HA-5
	G1153A	G374E	Genomewide	HA-16
	A1345C	N438T	HA/NA	HA-24
5 (NP)	A151U	I36F	Genomewide	NP-6
	U521C	M159T	Genomewide	NP-2
	C1194A	S383R	Genomewide	NP-4
	A1505G	Y487C	Genomewide	NP-10
6 (NA)	U334G	F105L	HA/NA	NA-24
	G422A	D135N	HA/NA	NA-13
	A449C	S144R	HA/NA	NA-25
	U668A	C217S	HA/NA	NA-31
	U693C	I225T	Genomewide	NA-8
	G968U	G317C	Genomewide	NA-6
	G1313U	V432L	Genomewide	NA-7
	U1340G	W441G	Genomewide	NA-2
	U1400C	Non-Coding	HA/NA	NA-28
7 (M)	U506C	M1 S161P	Genomewide	M-8
	G551A	M1 E176K	Genomewide	M-6
	G936U	M2 E75stop	Genomewide	M-3
	A1009G	Non-Coding	Genomewide	M-5

### Clonality of viable mutants

As in other studies of viral mutational fitness effects, we measured the fitness of P1 viral stocks rather than the initial transfection supernatant. Given the error prone replication of RNA viruses, we considered it possible that second-site mutations might accumulate in the short time between transfection and completion of the first passage. This might be exaggerated in the less fit viruses, since there would be strong positive selection of a compensatory mutation. If a compensatory mutation swept through the population, it would confound assignment of a fitness value to the initial mutation. Therefore, we sequenced the entire genome of the P1 stocks for 78 of our mutants on the Illumina platform to identify any second site mutations and their frequency. In all but 3 cases, the desired mutation was present at >94%, and was present at >99% in nearly all. Very few additional mutations were identified at >2% (Table 2, S2 Table). We relied on Sanger sequencing alone to confirm the desired mutation in replicate stocks of the remaining 13 viable viruses. We conclude that these stocks are essentially clonal and that the fitness of these populations will reflect the impact of each individual mutation.

**Table 2:** Next generation sequencing of viral stocks.

Mutant	Mutation	Amino Acid	Mutation > 95%	Secondary Mutations (frequency)	Dataset
HA-10	T1599A	S523T	Yes	PA-A95G (5.0)	Genomewide
HA-21	A334T	N101I	Yes *	- *	Genomewide
HA-30	A648C	S206R	94.7	HA-T1583G (4.6); HA-A534G (2.7)	HA/NA
HA-46	C231G	L67V	Yes *	- *	HA/NA
M-1	G661C	M212I	Yes	M-T117G (3.0)	Genomewide
M-7	C174G	C174G	Yes *	- *	Genomewide
NA-14	G98A	G27R	Yes	PB2-G2086T (2.5)	HA/NA
NA-20	A909C	K297T	Yes	PB2-T2156A (2.8)	HA/NA
NA-9	G1355A	G1355A	0.75	NS-C684T (2.7)	Genomewide
NP-8	A454C	A454C	Yes	PB2-A233G (2.5)	Genomewide
NS-2	G227T	R76R	Yes	PB2-A235G (2.7)	Genomewide
PA-1	C500T	A159V	92.6	PA-C500G (5.2); PB2-A913G (3.3); PB2-C1919T (2.9)	Genomewide
PA-6	G240A	L72L	92.5	-	Genomewide
PA-7	A1358T	Y445F	Yes	PB2-T1627C (3.7)	Genomewide
PB1-11	A2187T	R721S	Yes	PA-T1469C (2.0)	Genomewide
PB2-8	C839A	T271K	Yes *	- *	Genomewide

* Sanger sequence only

### Measurement of viral fitness

**Fig 1:**
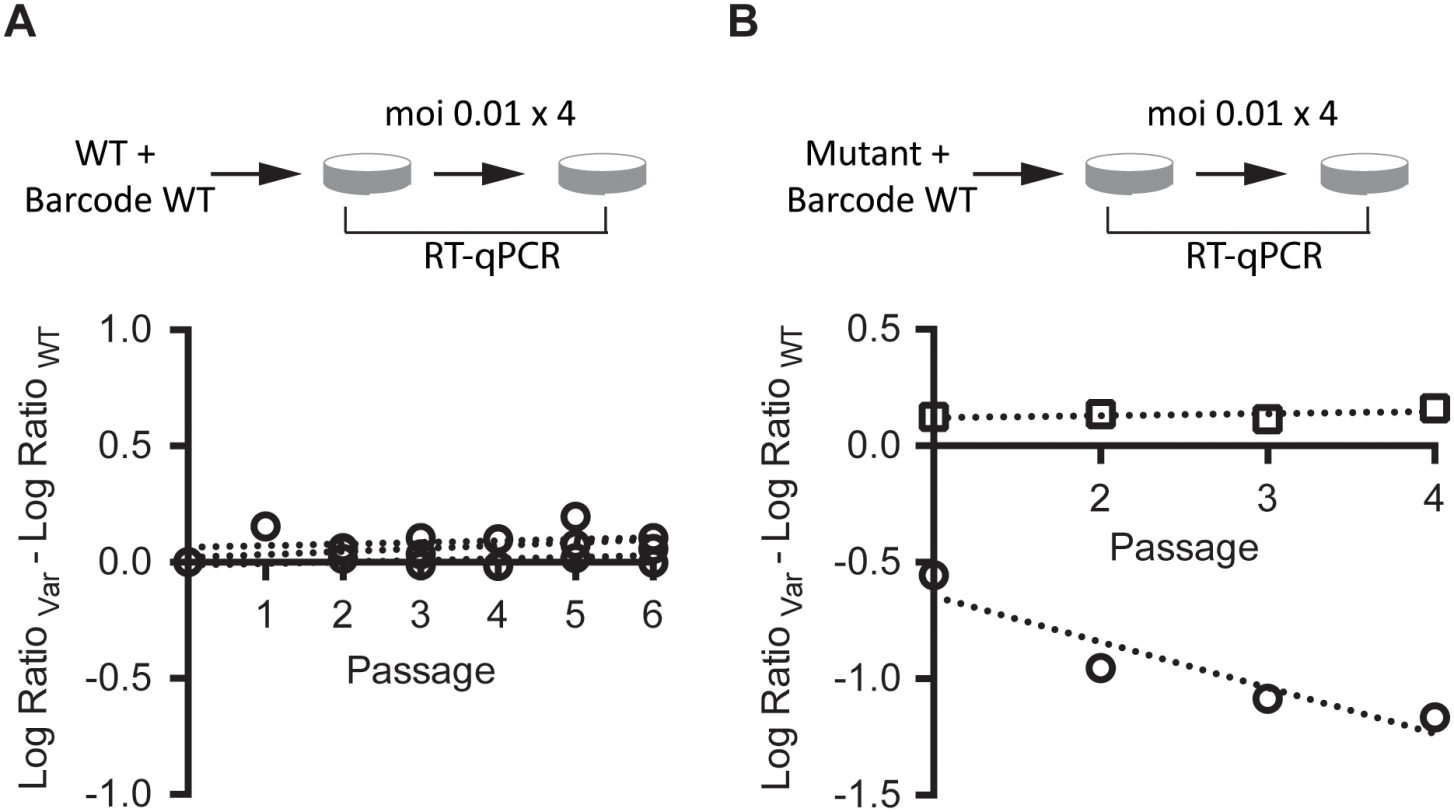
Direct competition assay for relative fitness. (A) Equal infectious units of a barcoded version of the WT were competed against WT at an moi of 0.01, and the amount of each virus at each passage was compared to the input by RT-qPCR as described in the methods. The slope of the regression of the difference in the log10 change in ratio for each virus over time is the fitness. The assay was performed in triplicate and the slopes of the three lines are 0.007, 0.129, and 0.007, which corresponds to a fitness of 1.02 ± 0.008 for the barcoded virus relative to WT. (B) Sample data for two single nucleotide mutants. Each was competed against the barcoded WT as in (A) and relative fitness measured as calculated in the methods. One replicate each of NA-18 (circles, fitness = 0.64) and HA-40 (squares, fitness = 1.02) is shown. Note that we fit our regressions through passages 1–4 and excluded P0 as slight deviations from a 1:1 ratio of the two viruses in the inoculum can skew the slope when fit through this data point.

In prior studies of MFE, fitness has been measured as the difference in exponential growth rates for WT and mutant strains, measured either in parallel or in direct competition[19,36,37,39,42]. Given the relative imprecision of one step growth curves for quantification of growth parameters in influenza and many other viruses, we measured the relative fitness of each mutant in direct competition with the WT over serial passage [18]. In this assay, the mutant is competed with a tagged WT reference, which has a cluster of synonymous mutations in the PB1 open reading frame. These mutations allowed us to distinguish the barcoded WT from a non-barcoded mutant in a mixed infection using quantitative reverse transcription PCR with primers specific for their respective sequences. Importantly, this tagged WT virus competed equally well with the untagged WT virus over 6 passages, demonstrating the selective neutrality of the marker (Fig 1A). In our serial passage competition assay, the change in relative frequency of the WT and mutant over time is the difference in growth rate, or the selection rate constant (Fig 1B). The exponent of this value is the relative fitness. We have shown previously that this assay can provide precise measurements of relative fitness with as few as 3 technical replicates [18], although it is less sensitive for weakly deleterious or beneficial mutations with a relative fitness close to 1.

We performed our competition assays in A549, a cancerous human lung epithelial cell line, which supported efficient replication of WSN33 and may be more physiologically relevant to influenza virus replication than other commonly used lines. Passages were carried out at a multiplicity of 0.01 infectious units (TCID_50_) per cell. Given that the burst size of influenza A in A549 cells is <100 per cell, most passages will represent 2 cellular infection cycles with minimal co-infection in the second cycle. We note that this moi corresponded to just 10,000 infectious units at each passage, and we cannot exclude that genetic drift could lead to some variability in the fitness measurements with a transfer population of this size [43].

### Distribution of mutational fitness effects

**Fig 2:**
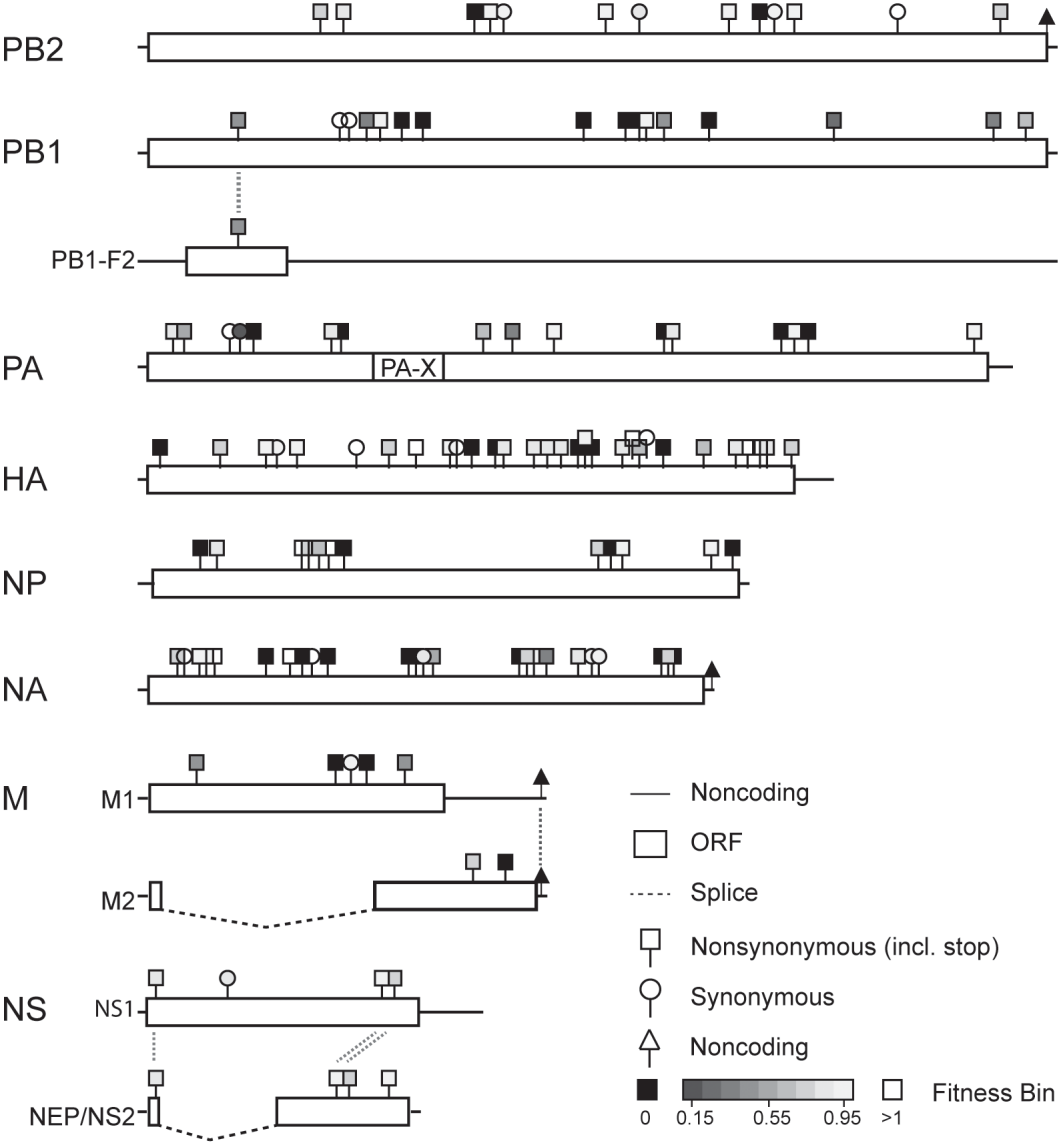
Location and fitness for all mutations. Each mutation in Tables 1 and 3 is shown in its reading frame(s) with substitution type (nonsynonymous, synonymous, or noncoding) and fitness (see legend).

**Fig 3:**
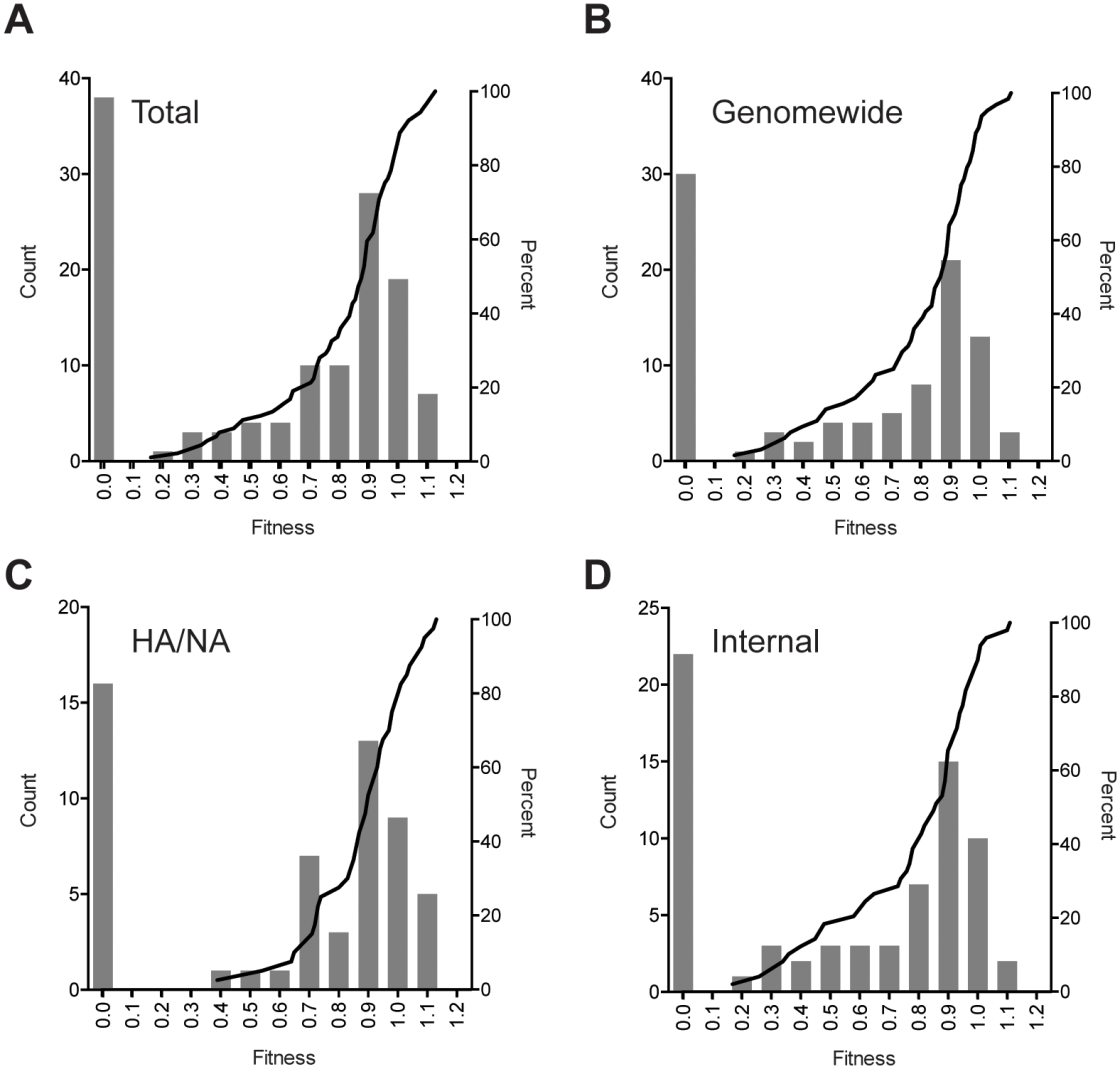
Histograms and cumulative distribution functions of influenza A virus mutational fitness effects. Data are shown for all single nucleotide mutants (A, n = 128), the randomly selected genome-wide dataset (B, n = 95), the HA and NA dataset (C, n = 57), and the “internal” dataset (D, n = 71). Relative fitness values, bin width 0.1, are shown on the x-axis, and number of mutations in each histogram bar (left) and percent in cumulative distribution (right) are shown on the y-axes. The cumulative distribution functions show only the viable mutations (fitness > 0).

We were able to measure the fitness of 89 out of the 90 viable mutants. Despite repeated attempts with multiple stocks, we were unable to obtain data for HA-8. In competitions with this mutant, we consistently saw large reductions in titer of both HA-8 and the wild type after a single passage, perhaps due to dominant negative effects or the impact of defective interfering particles. With the exception of PB1-6, which was measured twice, all fitness values are based on 3 replicate competition assays, and the mean fitness and standard deviation are reported in Table 3 (see also Figs 2 and 3). Seventy-one of the mutations in our library were nonsynonymous, and these viruses had relative fitness values ranging from 0.26 to 1.12 (mean 0.80, standard deviation 0.20). Nearly all of the 18 viruses with synonymous mutations exhibited a fitness close to 1 (mean 0.93, standard deviation 0.21). The sole exception was PA-6/G240A, which is synonymous in the canonical PA open reading frame (Leu72). Neither this mutation, nor any of the other PA mutations were within the alternate PA-X reading frame [44]. Three mutations affected proteins in two different reading frames: PB1 A251C (fitness 0.45), NS G648U (fitness 0.92), and NS G650C (fitness 0.78). Three out of the 4 nonsense mutations were lethal (see Table 1), the exception was NS-5/G648T (NS1 E208stop, NEP M50I), which had a fitness of 0.92. Consistent with the important role of 5’ and 3’ translated regions in RNA synthesis and packaging, 2 out of the 3 non-coding mutations were lethal [45]. The 3^rd^ was HA-8 (see above).

**Table 3:** Fitness values of viable mutants.

Segment	Mutation	Amino Acid Change	Fitness Mean	Fitness SD	Dataset	Clone ID
1 (PB2)	U306A	P93 (SYN)	0.90	0.13	Genomewide	PB2-16
	A440U	Q138L	0.78	0.18	Genomewide	PB2-12
	C532A	P169T	0.81	0.04	Genomewide	PB2-14
	C880U	H285Y	0.96	0.07	Genomewide	PB2-4
	A1167U	R380S	0.94	0.06	Genomewide	PB2-11
	U1251C	D408 (SYN)	0.89	0.05	Genomewide	PB2-3
	A1495C	S490R	0.96	0.18	Genomewide	PB2-8
	U1527A	R500 (SYN)	0.90	0.04	Genomewide	PB2-5
	G1660C	V545L	0.94	0.04	Genomewide	PB2-7
	A1854G	T609 (SYN)	1.03	0.05	Genomewide	PB2-15
	A2113C	I696L	0.73	0.14	Genomewide	PB2-13
2 (PB1)	A251C	PB1 D76A, PB1-F2 T45P	0.45	0.01*	Genomewide	PB1-6
	C522U	F166 (SYN)	1.10	0.15	Genomewide	PB1-2
	C549U	N175 (SYN)	1.01	0.24	Genomewide	PB1-1
	A581C	Q186P	0.30	0.01	Genomewide	PB1-10
	G599A	R192K	0.99	0.09	Genomewide	PB1-5
	U1288A	S422T	0.95	0.03	Genomewide	PB1-13
	A1322C	K433T	0.48	0.10	Genomewide	PB1-12
	G1764U	W580C	0.26	0.00	Genomewide	PB1-4
	A2187U	R721S	0.34	0.09	Genomewide	PB1-11
	A2277C	E751D	0.65	0.09	Genomewide	PB1-8
3 (PA)	A88G	K22E	0.81	0.12	Genomewide	PA-8
	A92G	E23G	0.58	0.05	Genomewide	PA-10
	U237A	L71 (SYN)	1.11	0.13	Genomewide	PA-9
	G240A	L72 (SYN)	0.17	0.07	Genomewide	PA-6
	C500U	A159V	0.85	0.11	Genomewide	PA-1
	U878G	M285R	0.62	0.14	Genomewide	PA-3
	U964G	F314V	0.36	0.13	Genomewide	PA-13
	G1041C	K339N	0.90	0.04	Genomewide	PA-2
	A1358U	Y445F	0.82	0.15	Genomewide	PA-7
	U1685C	I554T	0.89	0.08	Genomewide	PA-15
	U2123C	V700A	0.89	0.10	Genomewide	PA-16
4 (HA)	C231G	L67V	0.71	0.03	HA/NA	HA-46
	A334U	N101I	0.97	0.06	Genomewide	HA-21
	C368A	L112 (SYN)	0.93	0.08	Genomewide	HA-13
	U408A	S126T	0.95	0.16	HA/NA	HA-37
	G542U	K170N	1.01	0.12	HA/NA	HA-36
	A648C	S206R	0.72	0.14	HA/NA	HA-30
	A699U	N223Y	1.03	0.10	HA/NA	HA-38
	A784G	E251G	0.94	0.04	Genomewide	HA-22
	C822U	L264 (SYN)	1.00	0.06	HA/NA	HA-26
	A939U	N303Y	0.80	0.13	HA/NA	HA-41
	G1006U	S325I	0.83	0.08	HA/NA	HA-45
	A1050C	I340L	0.87	0.08	HA/NA	HA-43
	A1057U	Y342F	0.87	0.19	Genomewide	HA-1
	A1174U	Q381L	0.93	0.06	HA/NA	HA-27
	C1229G	I399M	0.85	0.10	Genomewide	HA-18
	A1264C	K411T	0.65	0.20	HA/NA	HA-31
	G1292A	M420I	1.12	0.07	HA/NA	HA-33
	U1299C	L423 (SYN)	1.13	0.27	HA/NA	HA-39
	U1466A	N478K	0.64	0.14	Genomewide	HA-3
	A1512G	S494G	0.87	0.04	HA/NA	HA-42
	U1583G	D517E	1.00	0.02	HA/NA	HA-40
	G1587C	V519L	1.06	0.24	Genomewide	HA-15
	U1599A	S523T	0.88	0.13	Genomewide	HA-10
	C1696A	S555Y	0.73	0.01	Genomewide	HA-20
	A1749C	noncoding	ND	ND	Genomewide	HA-8
5 (NP)	U198G	D51E	0.88	0.06	Genomewide	NP-1
	A425G	D127G	1.01	0.07	Genomewide	NP-9
	G436U	A131S	0.76	0.11	Genomewide	NP-5
	A454C	M137L	0.60	0.05	Genomewide	NP-8
	C485U	T147I	1.00	0.28	Genomewide	NP-7
	A1160U	E372V	0.77	0.10	Genomewide	NP-11
	A1229U	N395I	0.99	0.09	Genomewide	NP-12
	C1485A	D480E	0.99	0.04	Genomewide	NP-3
6 (NA)	G98A	G27R	0.73	0.03	HA/NA	NA-14
	C109A	I30 (SYN)	1.04	0.24	HA/NA	NA-30
	G158A	G47R	0.94	0.20	HA/NA	NA-22
	A176C	S53R	0.90	0.11	HA/NA	NA-21
	G201C	G61A	0.98	0.03	Genomewide	NA-4
	C454U	C145 (SYN)	1.08	0.11	HA/NA	NA-26
	C700U	T227 (SYN)	0.84	0.04	Genomewide	NA-10
	G758C	V247L	0.54	0.06	Genomewide	NA-3
	A909C	K297T	1.09	0.14	HA/NA	NA-20
	A1026U	K336M	0.39	0.08	HA/NA	NA-29
	G1040C	V341L	0.74	0.09	HA/NA	NA-18
	U1070G	S351A	0.90	0.19	Genomewide	NA-5
	U1130G	F371V	0.89	0.22	HA/NA	NA-16
	U1162C	T381 (SYN)	0.98	0.01	Genomewide	NA-1
	G1168U	R383 (SYN)	0.93	0.05	HA/NA	NA-19
	G1355A	E446K	0.71	0.06	Genomewide	NA-9
7 (M)	C174G	M1 P50R	0.40	0.11	Genomewide	M-7
	A541C	M1 L172 (SYN)	0.93	0.09	Genomewide	M-4
	G661C	M1 M212I	0.48	0.01	Genomewide	M-1
	U861G	M2 C50G	0.74	0.07	Genomewide	M-2
8 (NS)	U51A	NS1 F9I, NEP F9I	0.86	0.05	Genomewide	NS-1
	G227U	NS1 R67 (SYN)	0.85	0.13	Genomewide	NS-2
	G648U	NS1 E208stop, NEP M50I	0.92	0.04	Genomewide	NS-5
	G650C	NS1 E208D, NEP R51T	0.78	0.04	Genomewide	NS-4
	A809G	NEP Q104R	0.91	0.06	Genomewide	NS-3

ND, no data

* range of 2 replicates

### HA mutational fitness effects in head and stem regions

**Fig 4:**
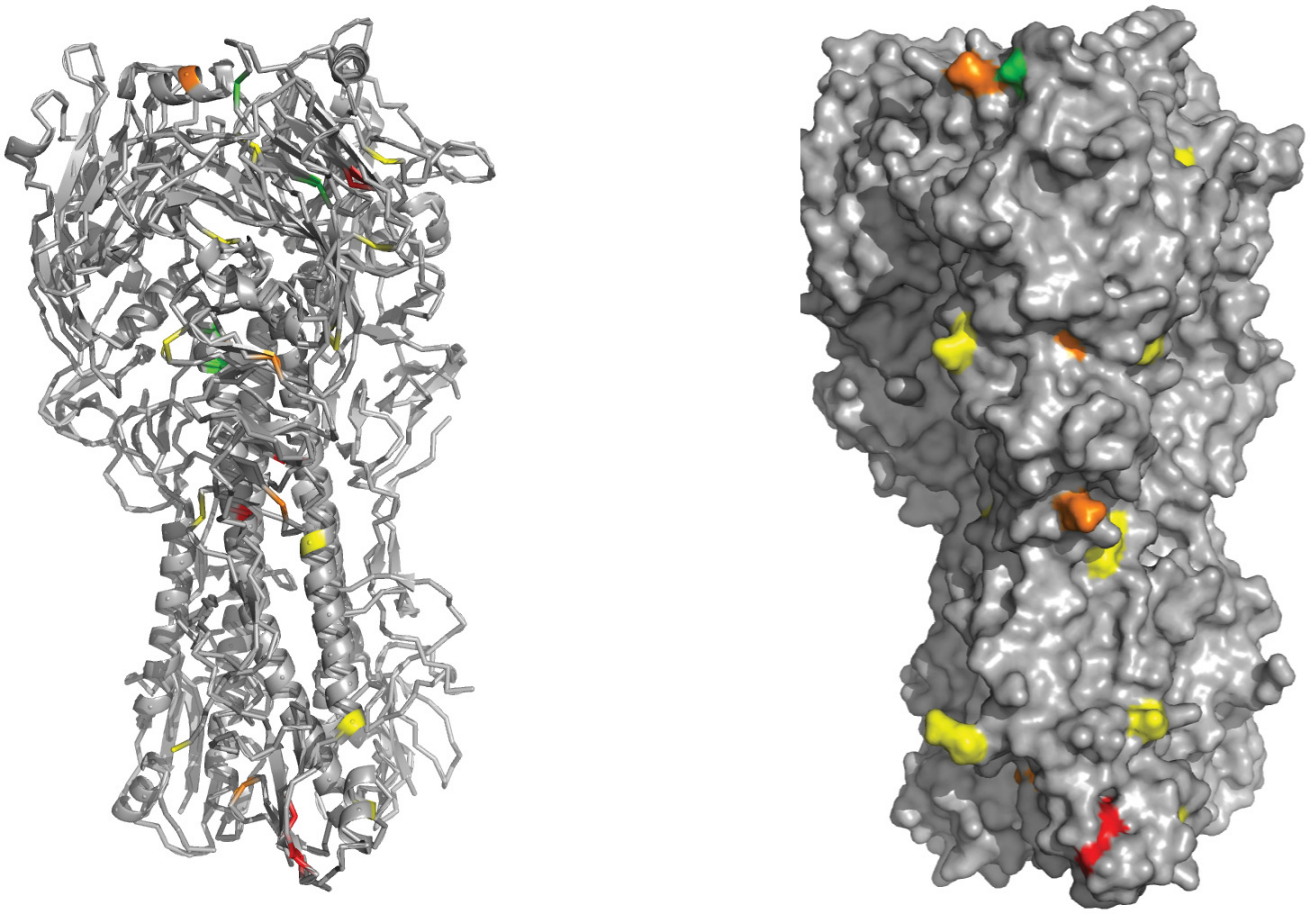
Fitness impact of mutations on HA. Mutations were placed onto a structural model of the hemagglutinin protein (PBD 1RVX). Shown are mutations on the head and stem regions, HA1 and HA2. Non-coding mutations (HA-8) and mutations on the signal peptide (HA-11), splice site (HA-1), and transmembrane domain (HA-40, HA-15, HA-10, HA-20) are not shown. Mutations are color coded as follows according to their relative fitness: lethal mutations are red, 0.6–0.8 are orange, 0.8–1.0 are yellow, and 1.0–1.2 are green. We found no HA mutations with a fitness between 0–0.6.

We mapped our HA mutants to the protein structure in order to observe structural patterns in HA fitness effects (Fig 4). Similar to previous studies on mutational fitness effects in influenza A hemagglutinin [35,46], we observed that the HA head seemed to tolerate mutations better than the stem domain. The mutants on the HA1 head domain had an average fitness of 0.77 and a lethal fraction of 2/14. Mutants on the HA2 stem region had an average fitness of 0.56 and a lethal fraction of 4/11. While suggestive, these differences in mean fitness did not achieve statistical significance (p = 0.258, Mann Whitney U test). Two mutations were located in one of the four H1N1 antigenic sites [47], K170N (Sa, fitness 1.01 ± 0.12) and S206R (Sb, fitness 0.72 ± 0.14).

### Fitting mutational fitness effects to probability density functions

We compared the distributions of fitness effects by plotting the fitness of all mutants as histograms and overlaying fitness values for the viable mutants as cumulative distribution functions. We show distributions for the total dataset (n = 128, Fig 3A), the randomly selected genome-wide dataset (n = 95, Fig 3B), the expanded HA/NA dataset (n = 57, Fig 3C), and the comparison “internal” 6 segment dataset (n = 71, Fig 3D). As above, the lethal fraction in each group was ~30%. Of the viable mutants, 47 out of 90 were weakly deleterious or neutral (fitness 0.85–1.05). Seven were weakly beneficial (fitness 1.05–1.15). We are conservative in classifying weakly deleterious and weakly beneficial mutations, since the fitness values for many of these mutants were not statistically different from 1. The mutations in 35 of the 90 viable mutants were more deleterious (fitness < 0.85). The mean and standard deviation for the viable mutants were: 0.82 ± 0.21 in the total, 0.80 ± 0.22 in the genome-wide, 0.88 ± 0.16 in the HA/NA, and 0.78 ± 0.24 in the internal datasets, respectively. While the HA/NA and internal datasets had a similar lethal fraction (Table 1 and associated text), the fitness impact in the viable fraction tended to be lower in the HA/NA-encoding segments relative to the internal segment group, but did not achieve statistical significance (p = 0.08, Mann Whitney U test).

The fitness values of the viable mutants were not distributed normally, and we therefore determined which type of distribution best fit the data. We modeled our empiric data on the deleterious mutations against exponential, gamma, beta, Weibull, and lognormal predictions and determined the best fit based on their Akaike information criteria (AIC, Table 4). For each dataset, the beta model was the best at capturing the distribution of fitness effects. We also fit distributions from a large, recently published dataset of poliovirus mutants [48]. Here, the beta model also provided the best fit. These beta models are described by two shape parameters, α and β, in which the expected value of the beta distribution is α/(α+β). The α and β parameters for each of the random, surface, and internal influenza MFE datasets as well as the poliovirus MFE dataset are similar (Table 4). Some have suggested that deleterious mutational fitness effects may be better explained by more complex models [49]. We therefore attempted to combine the above distributions with uniform distributions, in which we allowed the latter to describe a proportion of our mutants’ fitness effects. However, in the cases tested, adding this uniform distribution did not improve the fit of our models. While the influenza A and poliovirus mutational fitness effects were both best described by a beta distribution, there was no similar consensus among previously characterized viruses [19]. VSV was best described by a lognormal + uniform distribution, TEV by a beta distribution, phiX174 by an exponential distribution, QB by a gamma distribution, and F1 by a log normal distribution. In most of these fits, the AIC of the beta distribution did not differ appreciably from the AIC of the best-fit probability density function.

**Table 4:** Model fit for MFE distributions.

	Total	Genome-wide	HA/NA	Internal	Poliovirus
Exponential	109.824436	84.814139	49.11733	61.536311	6970.5079
Gamma	-2.633794	5.372353	-23.9851	9.75584	1338.4441
Weibull	-33.906937	-17.061899	-35.7708	-4.538429	-499.8882
Lognormal	12.427892	17.069145	-21.1132	18.0786	3174.4454
Beta	-68.630982	-48.693444	-42.91293	-28.713227	-2519.1795
Shape Parameters (95% CI)					
alpha	3.258 (2.277–4.487)	2.833 (1.882–4.062)	6.306 (3.531–10.216)	2.47 (1.544–3.715)	2.166 (2.086–2.247)
beta	0.985 (0.731–1.297)	0.893 (0.638–1.216)	1.398 (0.857–2.151)	0.88 (0.595–1.252)	1.022 (0.989–1.056)

### Fitness in alternate genetic backgrounds

All of our mutations were generated in the context of WSN33, a lab-adapted H1N1 strain. Recent work suggests that epistasis is quite prevalent across the influenza virus genome, and we therefore considered it likely that the same mutations would have distinct fitness effects in other genetic backgrounds [50–53]. To understand the degree to which our fitness measurements for specific WSN33 mutations are generalizable, we compared our data to those obtained for analogous mutants in WSN33 and other genetic backgrounds. Bloom and colleagues have used deep mutational scanning (DMS) to broadly sample most of the possible amino acid substitutions in the HA and nucleoprotein open reading frames [35,46,54,55]. Deep sequencing of mutant libraries before and after passage was used to infer site preferences for every amino acid at every site and to calculate site entropy, or inherent tolerance of substitution at a given site. Site entropy will tend to capture the general mutability of a position, while site preference is likely to be more specific for a mutation in a given genetic background. Importantly for our comparison, many of the plasmids had multiple mutations and the site preferences represent the average effect of a mutation in the background of very similar, but distinct, sequences.

**Fig 5:**
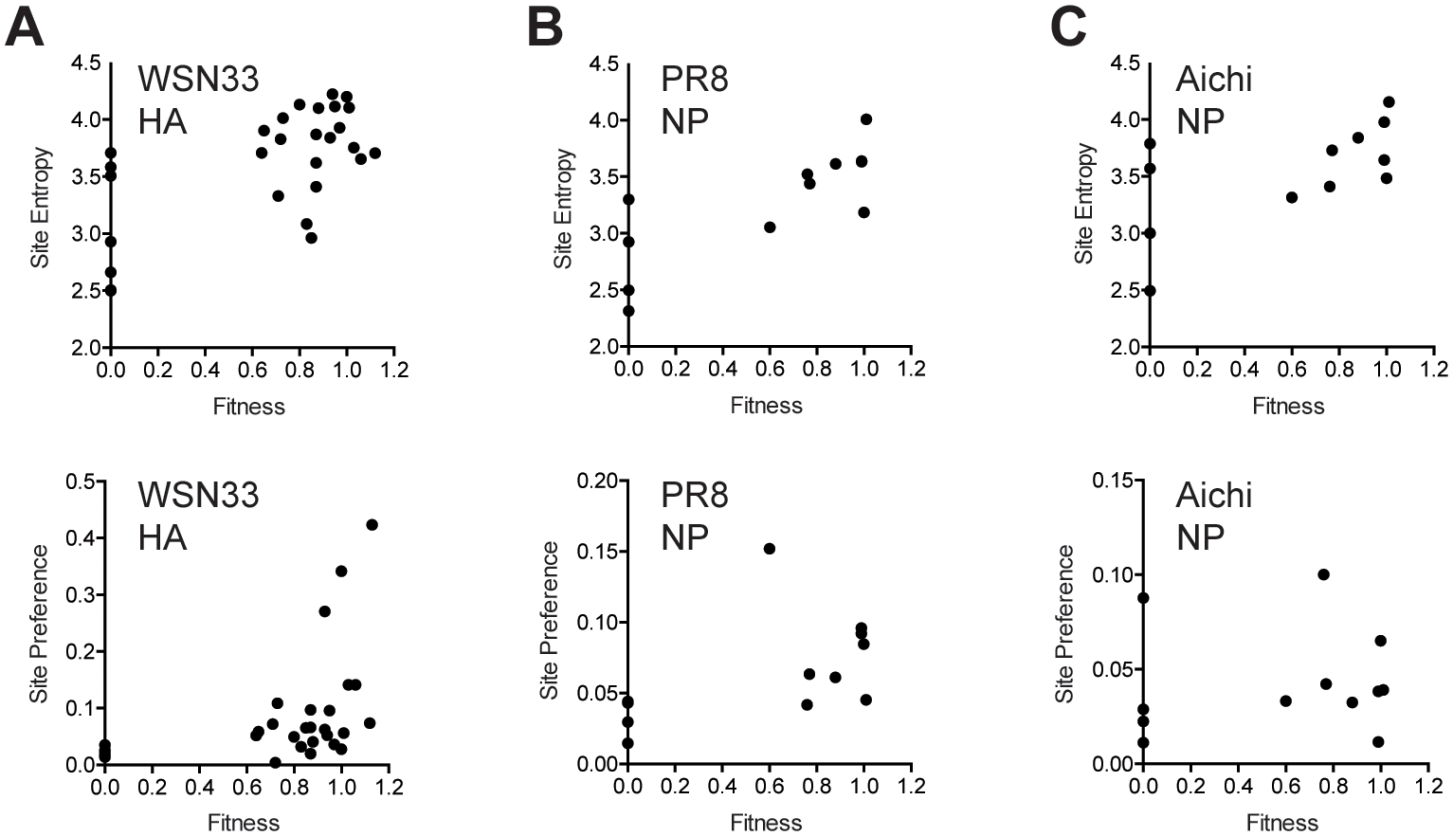
Correlation of fitness values with site entropy and preference. (A) Fitness of nonsynonymous HA mutants vs. site entropy (top) and fitness of all HA mutants vs. site preference (bottom) as reported in [46]. Unscaled values are shown. Correlations were similar for scaled values, see S3 Table. (B) Fitness of nonsynonymous NP mutants vs. site entropy (top) and fitness of all NP mutants vs. site preference (bottom) as reported for PR8 in [55]. (C) Fitness of nonsynonymous NP mutants vs. site entropy (top) and fitness of all NP mutants vs. site preference (bottom) as reported for A/Aichi/1968 (H3N2) in [55]. Note that scale in (A) for site preference is different as there were synonymous mutants in this dataset, but not in the NP datasets.

We first compared our 43 HA point mutants to DMS data on HA from WSN33 [35,46]. Here, we observed a statistically significant, and reasonably strong, correlation between our fitness values and site entropy for nonsynonymous substitutions (Fig 5A, Spearman r = 0.56, p = 0.0018). Consistent with the fact that both datasets were generated in the WSN33 background, the correlation between the fitness of viruses with our substitutions (nonsynonymous and synonymous) and the site preference for the same substitution was similar (Spearman r = 0.66, p < 0.001). It isn’t clear the degree to which the modest correlations for site entropy and site preference are attributable to differences in experimental set-up or the scale and error of measurement for two very different datasets. Of note, Thyagarajan and Bloom observed a similarly modest correlation (Pearson r = 0.48, p < 10^−10^) between their DMS data on WSN33 and a DMS separate study by Wu et al. [33], see discussion in [35].

We next compared the fitness of our 12 NP mutants to DMS data on NP from influenza A/Puerto Rico/8/1934 (H1N1, PR8) and A/Aichi/1968 (H3N2) [54,55]. In PR8, a closely related lab strain, we found a stronger correlation between fitness and site entropy (Fig 5B, Spearman r = 0.79, p = 0.0037). The correlation between WSN33 fitness and PR8 site preference was weaker (Spearman r = 0.60) but did achieve statistical significance (p = 0.0450). Interestingly, our WSN33 fitness values were correlated with site entropy in the more distantly related Aichi H3N2 strain (Fig 5C, Spearman r = 0.59, p = 0.0465). The correlation with site preference was weaker for the WSN33-Aichi comparison than the WSN33-PR8 comparison (Spearman r = 0.26) and did not achieve significance (p = 0.4031). Together, our fitness values are reasonably correlated with site entropy in distinct strains and subtypes, and these data suggest that a sizeable portion of mutational fitness effects are attributable to the general mutational tolerance at a given site. The observation that fitness was correlated with site preference for HA in WSN33 and NP in PR8 (H1N1), but not in Aichi (H3N2) suggests that the fitness impact of these mutations will vary with genetic background due to intragenic or intergenic epistasis. We are cautious in this interpretation given the differences in the underlying experimental design, sample size, and nature of the datasets, as outlined above. Given that Doud and Bloom found general conservation in site preference between PR8 and A/Aichi/1968, it is possible that our differences could be due to the type and number of amino acid substitutions in our smaller NP dataset.

We further explored the generalizability of our MFE data by querying the influenza research database for H1N1 sequences containing any of our nonsynonymous mutations. We reasoned that of our WSN33 amino acid substitutions, only those with weakly deleterious, neutral, or beneficial fitness effects would be observed in circulating strains. Indeed, we found only two mutations, both in HA, that were present at reasonably high frequencies in more recent, circulating strains (S4 Table, S2 Fig). One was weakly deleterious, Q381L (fitness 0.93 ± 0.06, present in 78.7%) and the other was beneficial, HA M420I (fitness 1.12 ± 0.07, present at 79.7%). Consistent with what one would expect, our deleterious mutations had extremely low prevalence in the database. The prevalence of only two other non-synonymous mutations was above 0.1%, HA N101I (fitness 0.97 ± 0.06, present in 0.247%) and NA D135N (lethal, present in 0.173%). The significance of these mutants in circulating strains is unclear as they represent a relatively small number of sequences in the entire database (54/21,805 and 28/16187, respectively).

### Comparison to other viruses

Sanjuan, Elena, and colleagues have used a similar approach to characterize the mutational fitness effects for 5 viruses: VSV, TEV, QB, phiX174, and F1 [13,19,37,40,56]. This set of viruses includes viruses with both DNA and RNA genomes (among which the authors found similar distribution patterns), and influenza is the first segmented virus to be described by this method. We compared the lethal fraction, mean, and variance of our mutational fitness effects to these previously characterized viruses. Because relative fitness values in VSV, QB, phiX174, and F1 were measured as differences in exponential growth rate, we first transformed our serial passage fitness values based on the wild type exponential growth rate in our experimental conditions (S5 Table). A similar transformation has been described for TEV to allow comparison of exponential growth rates across systems (see Methods and [19]). Overall, we found that the lethal fraction and our scaled fitness values closely matched those for these other small DNA and RNA viruses. We also calculated the skewness and kurtosis for each data set to quantitatively describe the shape of the distribution (Table 5). Following the general property that mutations are more likely to be deleterious than beneficial, we found negative values for skewness in each of our data sets. Kurtosis, measuring the “peakness” of a distribution, was above 5 for each of our data sets. A positive kurtosis value indicates that a probability density function has a heavier tail and more values near the mean than predicted by a Gaussian distribution.

**Table 5:** Comparison to MFE in other viruses.

	Genome	Sample	Lethal Fraction	Arithmetic Mean	Variance	Skewness	Kurtosis
VSV	ss (-) RNA	48	0.396	-0.132	0.036	-1.795	3.007
TEV	ss (+) RNA	66	0.409	-0.112	0.041	0.285	-0.382
QB	ss (+) RNA	42	0.286	-0.103	0.018	-1.167	0.238
phiX 174	ss DNA	45	0.200	-0.126	0.047	-1.957	4.022
F1	ss DNA	100	0.210	-0.107	0.037	-1.909	3.165
Influenza genomewide	seg -ssRNA	95	0.316	-0.124	0.027	-1.970	6.866
Influenza HA/NA	seg -ssRNA	57	0.281	-0.059	0.010	-1.790	7.101
Influenza internal	seg -ssRNA	71	0.310	-0.139	0.033	-1.728	5.557

## Discussion

We report the first genome-wide study of the mutational fitness effects of single nucleotide mutations in influenza A virus. Unlike other studies of mutational tolerance in influenza, we took great pains to define the lethal fraction, a key parameter in the distribution of MFE. We ensured the relative clonality of nearly all of our stocks by next generation sequencing and used a highly quantitative assay for fitness measurements. Both the lethal fraction and the overall distribution of MFE of influenza A are quite similar to what has been found for ssDNA and other RNA viruses. Consistent with what has been assumed, but to our knowledge never shown, the surface proteins of influenza virus appear to be slightly more tolerant of point mutation than the internal viral proteins. This finding did not achieve statistical significance. These results have important implications for quantitative models of influenza evolution and our general understanding of the mutational robustness of RNA viruses.

The MFE of many viruses were initially explored by mutation accumulation (MA) experiments involving serial plaque to plaque transfers [25–29]. While these studies suggested that many mutations were deleterious, MA experiments are generally unable to assign fitness values to individual mutations. Similarly, mutagen sensitivity has been used to define the global impact of mutation and the relative robustness of viral strains or even multiple viral species [23,30,31,57,58]. Neither gives a reliable estimate of the lethal fraction and comparisons across viral families can be confounded by differences in host cells, basal viral mutation rates, and the pleiotropic effects of commonly used mutagens on cellular metabolism. More recently, several groups have used higher throughput assays to measure viral mutational tolerance [33,35,48]. While these approaches allow for impressively large datasets, they are similarly imprecise in assigning fitness values to individual mutations. Because lethal and strongly deleterious mutations will be present at extremely low frequency, they are easily lost from populations with serial passage and variably ascertained by even the best deep sequencing approaches. For example, the impressive characterization of poliovirus MFE by CirSeq could only quantify the average fitness of a mutation across a range of haplotypes, given that each mutation can potentially arise in the setting of a genome that already bears a different mutation [48]. Many mutations were also present at very low frequency, varied significantly across passages, and were presumably subject to clonal interference. Measurement of fitness effects from these data also required extensive modeling of genetic drift. In influenza A virus, two different groups have used random mutagenesis of plasmids and next generation sequencing of recovered viruses to infer the mutational robustness of hemagglutinin [33,35] and nucleoprotein [54,55]. Because many of the plasmids had multiple mutations and the sequencing assays were unable to accurately measure fitness, these important works were nevertheless unable to model the distribution of MFE. The only genome-wide study of influenza A MFE, of which we are aware, utilized transposon-based insertional mutagenesis, and is less informative about the impact of single nucleotide mutation [59].

We chose a lower throughput, and in our opinion more reliable, approach to define the distribution of MFE in influenza A virus. Sanjuan, Moya, and Elena pioneered the use of small libraries of viruses with single nucleotide substitutions to model the MFE of vesicular stomatitis virus [13]. Since this initial study, Sanjuan has used a similar approach to define the MFE of the phages QB, f1, and phiX174 [36,37], and Elena has applied it to tobacco etch virus [40]. The advantage of this approach is that it links specific mutations to fitness values, provides precise fitness measurements, and accurately estimates the lethal fraction. We improved on this method by generating a larger genome-wide library of mutants and by also including sub-libraries to compare the impact of mutations on different viral proteins. Our exhaustive characterization of the lethal fraction by repeated transfection, documentation of the relative clonality of each stock by deep sequencing, and use of a highly quantitative competition assay also ensure the quality of our model of influenza A MFE. An obvious drawback of this approach is that it was only feasible to analyze 128 single nucleotide mutations. While we took great pains to ensure random selection of our mutations, it is possible that our random sample of mutations is biased in some way from the overall set of roughly 39,000 possible SNV in influenza A viruses. We are encouraged that our distribution is similar to what has been found in both high and low throughput studies [19,48].

Fitness values are specific to the environmental conditions in which they are measured [60]. For example, in cell culture they can vary with host cell type, temperature, and multiplicity of infection. We chose to use A549 cells, as they are derived from a respiratory epithelium and support relatively efficient replication of influenza virus. The variability and inefficiency of primary cells present difficulties for a large-scale comparative study such as ours. We chose a relatively low multiplicity to reduce bias in fitness measurements due to complementation and reassortment in multiply infected cells. We note, however, that our relatively low transfer populations in the serial passage experiment could contribute to the observed variability in replicate fitness measurements through genetic drift [43]. While our cell-based assay will capture elements of fitness related to virus interactions with the host cell machinery and evasion of the innate immune response, it does not model the impact of adaptive immunity. While we would argue that many of the deleterious mutations are likely to be so in many environments, some mutations that are only weakly deleterious in cell culture may be subject to stronger purifying selection in nature. Finally, our mutations in WSN33 may not have the same fitness impact in H3N2, or other subtypes, due to intragenic and intergenic epistasis. The observed correlation between site entropy and fitness on a set of a HA and NP mutants suggests that the overall distribution of MFE will be conserved.

Across viral systems, increased genetic variability is often observed in surface or structural proteins relative to internally located, non-structural proteins. This bias could be attributable to local differences in mutation rate or mutational robustness and may have implications for evasion of host antibody [61]. It is therefore interesting that the fitness impact of mutations in the segments coding for hemagglutinin and neuraminidase appear to be slightly less than those in proteins encoded by the other six genomic segments. Host-specific evolutionary rates are also higher for HA and NA relative to the other 6 segments [62]. The 6 internal segments have similar rates, and we were underpowered to distinguish differences in robustness among them. Our finding of a trend toward increased mutational tolerance in the HA protein, and the head in particular, is consistent with the tolerance of HA to transposon insertion [59] and deep mutational scanning experiments [35]. These data suggest that HA is more robust to mutation, possibly as a consequence of this history of strong and repeated selection for antigenic escape. A byproduct of this robustness could be the greater exploration of antigenic sequence space and subsequent immune escape [63]. Here, mutational robustness would increase evolvability, or the capacity for adaptive evolution. This concept is attractive in view of the proposed epochal evolution of influenza A H3N2 on neutral networks [64]. We note that our study was of an H1N1 virus, and there may be important differences in the mutational robustness of H3N2 and H1N1 surface proteins. This is especially important given their different evolutionary rates [65]. As we and others have pointed out, the relationship between mutational robustness and evolvability is complex, and there are clearly situations in which increased robustness will either increase or decrease evolvability [16,66–68].

The genome-wide distribution of mutational fitness effects of influenza A virus is similar to those of poliovirus ([48] and this work), VSV, tobacco etch virus, and phages F1, QB, and phiX174 [19,36–40]. Because we relied on an assumption of exponential growth over the course of the competition assay to transform our fitness values into exponential growth rates (see Methods), it is possible that we have either over- or underestimated the similarities. Given that this collection includes a negative sense ssRNA virus (VSV), a segmented negative sense RNA virus (influenza A), three positive sense ssRNA viruses, and two ssDNA viruses, it is clear that the type and polarity of the genomic nucleic acid are not major determinants of robustness. Their similarity likely reflects shared constraints due to small and compact genomes [19]. Small genome viruses often have overlapping reading frames or regulatory elements that are more sensitive to genetic disruption. Larger genomes allow for more genetic redundancy and modularity, which could limit the impact of point mutations. Interestingly, the hypersensitivity of these viruses to mutation at an individual level may actually promote robustness at a population level [69]. In large populations under strong purifying selection, deleterious variants are rapidly purged and the wild type sequence preserved. We note that recombination and reassortment in RNA viruses are often considered to be adaptive as they could also increase robustness by purging deleterious mutations from populations [70–72]. Given the association between sex and mutation fitness effects in evolutionary theory, it is interesting that we find similar robustness of an asexual virus (VSV), a recombining virus (poliovirus) and a reassorting virus (influenza). While our assay conditions minimized reassortment, a long-term life history of reassortment does not appear to have altered the intrinsic mutational fitness effects of influenza virus.

The evolutionary dynamics of influenza A virus have been intensively studied at the global and molecular level. Our data on mutational fitness effects will be useful in modeling these processes across scales. We expect that further comparative studies in this area might elucidate important similarities and differences between different influenza subtypes and between influenza and other viruses.

## Materials and Methods

### Viruses and cells

Madin Darby canine kidney cells (MDCK) were provided by Dr. Arnold Monto (University of Michigan) and A549 human lung epithelial cells were provided by Dr. Michael Bachman (University of Michigan). Human embryonic kidney 293T fibroblasts were provided by Dr. Raul Andino (UCSF). Both cell lines were maintained in Dulbecco’s Modified Eagle Medium (DMEM, Invitrogen) supplemented with 10% fetal bovine serum (Gibco and HyClone), 25mM HEPES (Invitrogen), and 0.1875% bovine serum albumin (Life Technologies). Viral infections were performed in DMEM supplemented with 25mM HEPES, 0.1875% bovine serum albumin, and 2μg/ml TPCK-trypsin (Worthington). The 8 plasmid reverse genetic system containing the genomic segments for influenza A/WSN33 (pHW181-PB2, pHW182-PB1, pHW-183-PA, pHW184-HA, pHW185-NP, pHW186-NA, pHW187-M, pHW188-NS) was a kind gift of Robert Webster ([41], St. Jude’s Children’s Research Hospital)

### Site directed mutagenesis

An R script was used to select randomly the nucleotide position and base change for all mutants in this study and to design optimal oligonucleotides for mutagenesis by polymerase chain reaction (PCR). Noncoding and promoter regions were included. Due to sequence context and GC content, several primers had to be designed manually (S1 Table). One hundred and eight of the single nucleotide mutants were generated using the QuickChange site directed mutagenesis kit (Agilent Technologies) according to the manufacturer’s protocol. The remaining 21 single nucleotide mutants were generated by overlap extension PCR [73]. Here, the outer primers contained 5’ BsmBI or BsaI restriction sites for subsequent cloning into pHW2000 [41,74]. The barcoded PB1 segment (555) was made using the Quickchange protocol and primers pPolPB1_555f 5’ GATCACAACTCATTTCCAACGGAAACGGAGGGTGAGAGACAAT 3’ and pPolPB1-555r ATTGTCTCTCACCCTCCGTTTCCGTTGGAAATGAGTTGTGATC. In each plasmid clone, the presence of the desired mutation(s) and the absence of second site mutations were verified by sequencing of the entire influenza segment.

### Transfection and viral stocks

Equal quantities of MDCK and 293T cells were seeded at a total density of 1,000,000 cells per well of a 6 well plate 24 hours prior to transfection in complete DMEM (see above). Each transfection mixture contained 1μg of the mutant plasmid, 1μg of each of the other 7 wild type plasmids, 16μl of TransIT-LT1 (Mirus) and 250μl Optimem (Gibco). These reagents were incubated together for 45 minutes at room temperature and applied dropwise to the cellular monolayer. After 24 hours, the media was changed to viral infection media (see above). Recombinant passage 0 virus was harvested 48 hours post-transfection, clarified by centrifugation at 200 x g for 3 minutes, and stored in aliquots with 0.5% glycerol at minus 70°C. Passage 1 (P1) stocks were generated by a single passage on MDCK cells at a multiplicity of infection (moi) ≤ 0.001. Virus was applied to cells for one hour and aspirated, and then viral media was added. Passage 1 stocks were harvested at 48 hours post-infection. All stocks were titered by tissue culture infectious dose (TCID_50_, [75]). We subjected all transfection supernatants with undetectable P0 titers to blind passaging. One or 0.333 ml of virus was applied to a confluent T75 or T25 flask, respectively. These cultures were monitored for cytopathic effect and titered at 48 hours.

### Next generation sequencing

Viral RNA was harvested from 200μl of each P1 supernatant using either Purelink Viral RNA (Invitrogen) or Qiamp Viral RNA (Qiagen) kits. Multiplex reverse transcription-PCR amplification of all 8 influenza virus genome segments was performed on RNA samples using Superscript III with HiFi platinum Taq (Invitrogen 12574) and primers Uni12/Inf1 (5’-GGGGGGAGCAAAAGCAGG-3’), Uni12/Inf3 (5’-GGGGGAGCGAAAGCAGG-3’), and Uni13/Inf1 (5’-CGGGTTATTAGTAGAAACAAGG-3’) [76]. Seven hundred fifty nanograms of the each amplified cDNA were sheared to an average size of 300 to 400 bp using a Covaris S220 focused ultrasonicator. Sequencing libraries were prepared using the NEBNext Ultra DNA library prep kit (NEB E7370L), Agencourt AMPure XP beads (Beckman Coulter A63881), and NEBNext multiplex oligonucleotides for Illumina (NEB E7600S). Indexed samples were pooled in equal quantities and sequenced on an Illumina MiSeq instrument with 2 x 250-base paired end reads.

Sequencing reads that passed standard Illumina quality control filters were binned by index and aligned to the reference genome using bowtie [77]. Single nucleotide variants (SNV) were identified and analyzed using DeepSNV [78]. The DeepSNV algorithm relies on a clonal control to estimate the local error rate within a given sequence context and to identify strand bias in base calling. It then applies a hierarchical binomial model based on mutation calls for test and control at each base and position to identify true-positive SNV. The clonal control was a library prepared in an identical fashion from 8 plasmids containing the A/WSN33/H1N1 genome and sequenced in the same flow cell. Code for our implementation of DeepSNV can be found at https://github.com/lauringlab/variant_pipeline, and can be accessed anonymously. True positive SNV were identified from the raw output tables (S2 Table) by applying the following filtering criteria in R: (i) Bonferonni corrected p value < 0.01, (ii) average MapQ score on variant reads > 30, (iii) average phred score on variant positions > 35, (iv) average position of variant call on a read > 50 and < 200, (v) variant frequency > 0.02. Application of these criteria to a viral spike-in dataset with equivalent genome copy number inputs resulted in > 95% sensitivity and > 99.99% specificity for SNV present at > 1% of the population [79].

### Competition assays

Competitions were performed on A549 cells in 12 well plates, plated at a density of 2.6 x 10^5^ per well 24 hours prior to infection. Cells were infected at a total MOI of 0.01 with an equal TCID_50_ of WT and a given mutant. Three replicate wells were infected with each pair of viruses. Passage 1 virus was harvested after 48 hours and one replicate was titered by TCID_50_. This titer was used to calculate the dilution factor necessary to maintain an MOI of 0.01 for subsequent passages. Four passages were performed for each competition. RNA was harvested from each passage using PureLink 96 well RNA mini kits (Invitrogen). Random hexamers were used to prime cDNA synthesis with 1/10 of the RNA. Each cDNA was analyzed by real time PCR using three different primer sets with duplicate PCR reactions for each sample/primer set. The first set, PB1_149f 5’ CAGAAAGGGGAAGATGGACA 3’ and PB1_360r 5’ GTCCACTCGTGTTTGCTGAA 3’, were used to quantify total viral genomic RNA. The second set, pPol1PB1_555f 5’ TCAGAGAAAGAGACGAGTGAG 3’ and pPol1PB1_555r 5’ AAACCCCCTTATTTGCATCC 3’, were used to quantify the amount of mutant viral RNA. The third set, pPol1PB1_555fm 5’ ATTTCCAACGGAAACGGAGGG 3’ and pPol1PB1_555r 5’ AAACCCCCTTATTTGCATCC3’, were used to quantify the amount of barcoded WT viral RNA. We verified that the levels of RNA (Cycle threshold, Ct) were well correlated with the infectious titer (TCID_50_/ml) for the mutants shown in Fig 1B. Duplicate wells were averaged and values were excluded from subsequent analysis if duplicates wells differed by > 0.5 Ct or any of the Ct were out of the empirically determined linear range for that primer pair. Relative amounts of WT and mutant RNA were determined by normalizing the cycle thresholds for each to those of the common (PB1_149f and PB1_360r) primer set (DCt = Ct_Virus_-Ct_PB1 total_). The normalized values for each virus passages 1–4 were then compared to passage 0 to obtain a ratio relative to P1 (ΔΔCt = Ct_PX_-Ct_P0_). This relative Ct value was converted to reflect the fold change (Δratio = 2^-logΔΔCt^). The change in ratio of the mutant relative to the change in ratio of the WT as a function of passage is the fitness ([Δratio_Mut_-Δratio_WT_]/time).

### Transformation of fitness into relative growth rate

Except for poliovirus, the data on other viruses were extracted from [19]. Here, the relative fitness values for VSV, QB, phiX174, and F1 were calculated as differences in the exponential growth rate. The data on TEV were originally derived by qPCR as in the present study. In this comparative analysis, Sanjuan applied a correction to the TEV data to enable direct comparisons of growth rate. We applied this same correction to our data to transform the values to something resembling an exponential growth rate. The exponential growth rate, r, of influenza A on A549 in a 12 well dish at moi 0.01, is derived from the equation: N_t_ = N_0_e^rt^, where t = 2 days (48 hour passage), N_0_ is 10,000 infectious units (the amount at time 0), and N_t_ is the titer after time t, or 48 hours. For N_t_, we used the mean of three replicates for HA-40 (9.3e5), which has a fitness of 1 (see Fig 1B). This is similar to the scaled output of WSN33 that we have observed at different moi and different well sizes. Solving for r gives a value of 2.26 per day. We then applied the following correction from [19], y = (lnx + r)/r, where y is the relative exponential growth rate of a given mutant, x is the relative fitness measured by qPCR and reported in Tables 1 and 3, and r is the estimate of the WT exponential growth rate under our experimental conditions (2.26 per day). For example, a fitness of 0.8 in our assay is transformed to a relative exponential growth rate of 0.90. The relative exponential growth rates, as calculated, are reported in S5 Table. We note that using a value 2e6 for N_t_ (which would be roughly consistent with the average dilution factor applied at each passage in many of the competition experiments, gives a growth rate of 2.65 per day and relative exponential growth rate of 0.916 for a virus with a fitness of 0.8.

### Statistics

All secondary analysis and statistical tests were performed in R and GraphPad Prism6. R and Python scripts for analysis of Influenza Research Database data (as of July 8, 2016) and other analyses are available at https://github.com/lauringlab/MFE_paper.

## Supporting Information

S1 Fig Distribution of single nucleotide variants in the influenza A genome. (A) A randomly selected genome-wide dataset (n = 95), in which the number of mutants generated per segment (grey bars) closely matches the expected distribution based on percentage of the genome contained on each segment (black bars). (B) Extra HA and NA mutations were generated to compare larger numbers of mutations on these segments, n = 57, to those on the other 6 (internal) segments, n = 71.
(PDF)

S2 Fig Frequency of mutations in the Influenza Research Database. Shown are the frequencies of nonsynonymous amino acid substitutions in our mutant dataset (y-axis) and their fitness values (x-axis).
(PDF)

S1 Table Primers used in this study. (XLSX)

S2 Table Secondary mutations as determined by Illumina sequencing. (XLSX)

S3 Table Site entropy and site preference values for HA and NP mutants. (XLSX)

S4 Table Raw data on mutation frequency from the Influenza Research Database. (XLS)

S5 Table Relative exponential growth rates for all mutants. (XLSX)
