# Supplementary material for: The Mutational Robustness of Influenza A Virus
Source: PLoS Pathog. 2016 Aug 29;12(8):e1005856. doi: 10.1371/journal.ppat.1005856 (PMC5003363; doi:10.1371/journal.ppat.1005856)
Supplement: S1 Fig — (A) A randomly selected genome-wide dataset (n = 95), in which the number of mutants generated per segment (grey bars) closely matches the expected distribution based on percentage of the genome contained on each segment (black bars). (B) Extra HA and NA mutations were generated to compare larger numbers of mutations on these segments, n = 57, to those on the other 6 (internal) segments, n = 71. (PDF) [file ppat.1005856.s001.pdf]

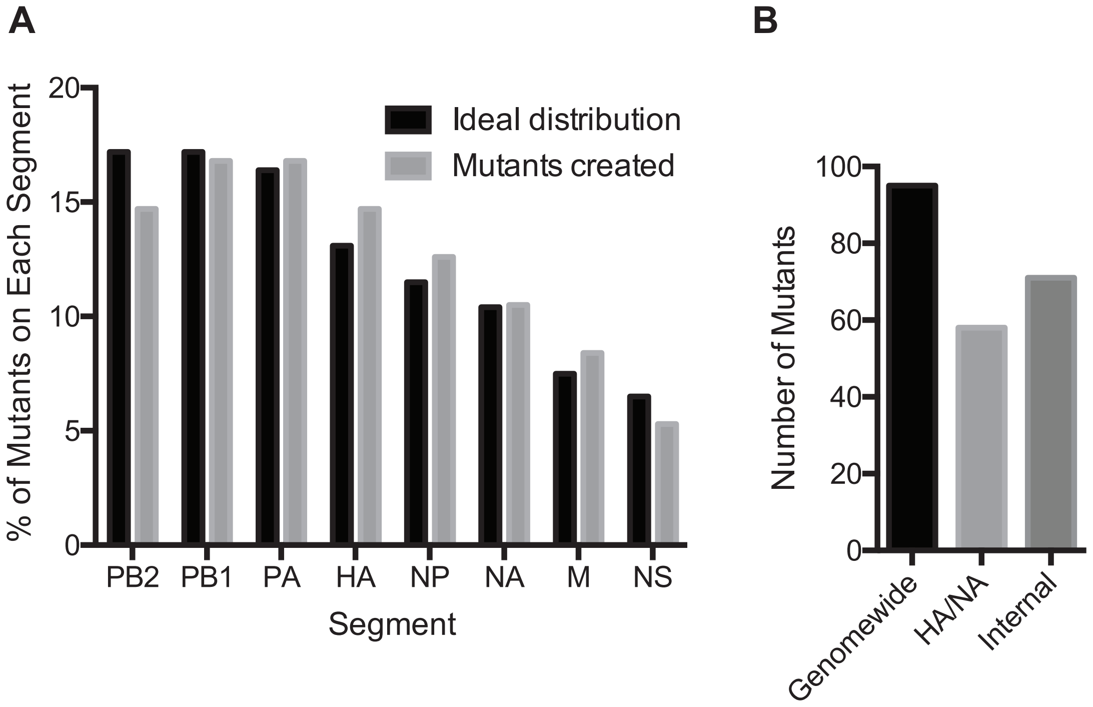

Figure S1: Distribution of single nucleotide variants in the influenza A genome. (A) A randomly selected genome-wide dataset (n=95), in which the number of mutants generated per segment (grey bars) closely matches the expected distribution based on percentage of the genome contained on each segment (black bars). (B) Extra HA and NA mutations were generated to compare larger numbers of mutations on these segments, n=57, to those on the other 6 (internal) segments, n=71.
