# Supplementary figures and images for: The Mutational Robustness of Influenza A Virus
Source: PLoS Pathog. 2016 Aug 29;12(8):e1005856. doi: 10.1371/journal.ppat.1005856 (PMC5003363; doi:10.1371/journal.ppat.1005856)

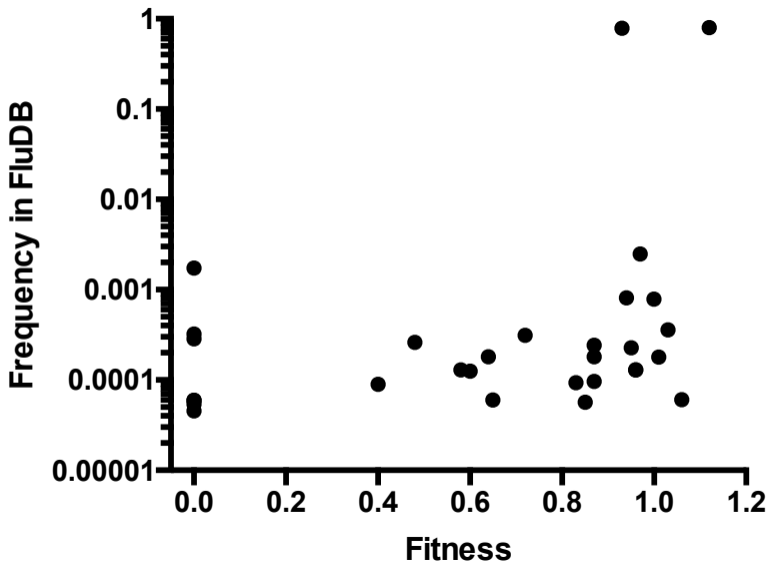

Supplement: S2 Fig — Shown are the frequencies of nonsynonymous amino acid substitutions in our mutant dataset (y-axis) and their fitness values (x-axis). (PDF) [file ppat.1005856.s002.pdf]
